# Recent Advances in Unveiling the Role of Beta-Herpesviruses in Autoimmune Diseases

**DOI:** 10.3390/microorganisms9122572

**Published:** 2021-12-13

**Authors:** Maria Cristina Arcangeletti, Elisabetta Caselli

**Affiliations:** 1 Department of Medicine and Surgery, University of Parma, 43126 Parma, Italy; mariacristina.arcangeletti@unipr.it; 2 Section of Microbiology, Department of Chemical, Pharmaceutical and Agricultural Sciences and LTTA, University of Ferrara, 44121 Ferrara, Italy

A dysregulated immune response can lead to recognition of self-antigens as non-self-antigens, leading to failure of the immune tolerance toward normal cells and tissues, and the consequent development of a variety of autoimmune diseases. Such diseases stem from a complex interaction between factors including the genetic background and environmental factors which can act as triggers of the dysregulated immune response, finally leading to tissue attack and loss of function or direct destruction.

Autoimmune diseases affect up to 5% of the general population and include more than 80 chronic and progressive diseases, most of which are without recognized causes and specifically targeted therapies [1].

Among environmental triggers, herpesvirus infection is reportedly associated with the development of autoimmunity, including alpha- (Herpes simplex type 1, HSV-1), beta- (Human cytomegalovirus, HCMV, and Roseoloviruses), and gamma-herpesviruses (Epstein Barr virus, EBV) [2,3,4,5,6], based on their frequent presence in the affected patients and on the detection of specific and/or dysregulated immune response toward them [7].

In particular, beta-herpesvirus sub-family, including Human Cytomegalovirus (HCMV) and the Roseoloviruses Human herpesvirus-6A (HHV-6A), -6B (HHV-6B), and -7 (HHV-7), includes ubiquitous viruses with a long double strand DNA genome encoding more than 200 open reading frames, some of which produce factors that are able to directly interact with and modulate the immune response. Indeed, they have evolved an arsenal of immunomodulatory proteins and miRNAs, with several genes that are homologous to cell genes, most of which affect immune-related processes or controlling immune cell apoptosis [8]. All of them establish lifelong infections in the host, thanks to their ability to persist in a latent phase and eventually reactivate in specific cells.

These characteristics, together with their wide cell/tissue tropism, render beta-herpesviruses good candidates as etiological agents of autoimmune diseases. However, a causative link between virus infection and autoimmune disease onset has not been conclusively established yet, nor the research has clarified whether those viruses are initiators of autoimmune diseases or simply bystanders, exacerbating the course of the pathology.

In addition, beta-herpesviruses have mutualistic relationships and often are found co-reactivated in the host [7,9,10,11]; co-reactivation is associated with worse clinical outcome.

Recently, the putative role of HCMV and HHV-6 infection has been investigated in autoimmune diseases, such as systemic sclerosis [12,13,14,15], multiple sclerosis [16,17], systemic lupus erythematosus [18], and Hashimoto’s Thyroiditis [19].

Nevertheless, though research studies have put light on several aspects of the beta-herpesvirus replication and potential pathogenesis, no studies on the possible cooperation between beta-herpesviruses have been accomplished, nor has a clear-cut role been established for each individual virus in any of the hypothesized associated autoimmune diseases.

Furthermore, being beta-herpesviruses considered poorly pathogenic viruses, the research program historically suffered from underfunding to the benefit of other more virulent viruses or more urgent pathologies. For instance, beta-herpesvirus infection could also play a role in the current viral pandemic in relation to the COVID-19 severity in patients with autoimmune diseases. In fact, a higher prevalence of symptomatic SARS-CoV-2 infection has been detected among patients with autoimmune systemic diseases, such as systemic sclerosis [20]. In critically ill patients with COVID-19, a significantly high incidence of HCMV and HHV-6 reactivation was observed [21,22,23].

In this issue, the contributions from leading authors may provide:

- A comprehensive update on the status of the research on beta-herpesvirus and autoimmune diseases;
- Insight into serum or tissue markers of beta-herpesvirus infection;
- Studies highlighting the implementation of immunological and functional assays to understand the role of beta-herpesviruses in the etiopathogenesis of autoimmune diseases;
- Studies describing methods which can be used to distinguish HHV-6A from HHV-6B responses.

The impact of deepening our understanding of the mechanisms by which beta-herpesvirus can switch on the onset or the development of autoimmune diseases may help to reduce the risk to contract, slow, or block the progression of such diseases, opening the way for new, advanced therapies that are targeted to virus replication, viral products, or virus-induced factors inside the body.

Taken together, this collection of reports will help to improve such understanding, ultimately expediting the design of appropriate diagnostic and therapeutic tools.
